# Primary extramedullary plasmacytoma of the sigmoid colon with perforation: a case report

**DOI:** 10.1186/s40792-018-0437-0

**Published:** 2018-04-04

**Authors:** Fumimasa Kitamura, Koichi Doi, Hiroyuki Ishiodori, Tetsufumi Ohchi, Hideo Baba

**Affiliations:** 1 Department of Gastroenterological Surgery, Nobeoka Hospital, 2-1-10 Shinkoji, Nobeoka, Miyazaki, 882-0835 Japan; 2 Department of Gastroenterological Surgery, Graduate School of Medical Science, Kumamoto University, Kumamoto, Japan

**Keywords:** Extramedullary plasmacytoma, Colon neoplasms, Perforation

## Abstract

**Background:**

Extramedullary plasmacytomas account for 4% of all plasma cell tumors and occur mainly in the upper respiratory tract; gastrointestinal system involvement is rare. Extramedullary plasmacytoma of the colon with perforation has not been reported.

**Case presentation:**

A 77-year-old woman with a 1-year history of lower abdominal pain and nausea was admitted to our hospital. An abdominal computed tomography scan revealed a sigmoid tumor with perforation. The patient underwent emergency surgery. Pathological examination led to a diagnosis of plasmacytoma of the colon. The patient did not undergo postoperative adjuvant chemotherapy. She has had no recurrence in 14 months of regular follow-up.

**Conclusions:**

We have herein described a rare case of extramedullary plasmacytoma of the gastrointestinal tract with perforation involving the sigmoid colon.

## Background

A plasma cell tumor is an immunoproliferative monoclonal disease of the B cell line that originates from malignant transformed plasma cells. Plasmacytoma includes solitary plasmacytoma of bone and solitary extramedullary plasmacytoma.

Solitary extramedullary plasmacytoma has been rarely reported, and its natural history and diagnosis are unclear. Most such plasmacytomas occur in the nasopharynx or upper respiratory tract; only 10% of reported cases have involved the gastrointestinal tract. The stomach and small intestine are the most commonly involved sites in the gastrointestinal tract [1–3]. Primary isolated extramedullary plasmacytoma of the colon is extremely rare. No previous reports have described plasmacytoma of the colon with perforation. We herein report a rare case of primary isolated extramedullary plasmacytoma of the colon with perforation and describe the patient’s postoperative clinical course.

## Case presentation

**Fig. 1:**
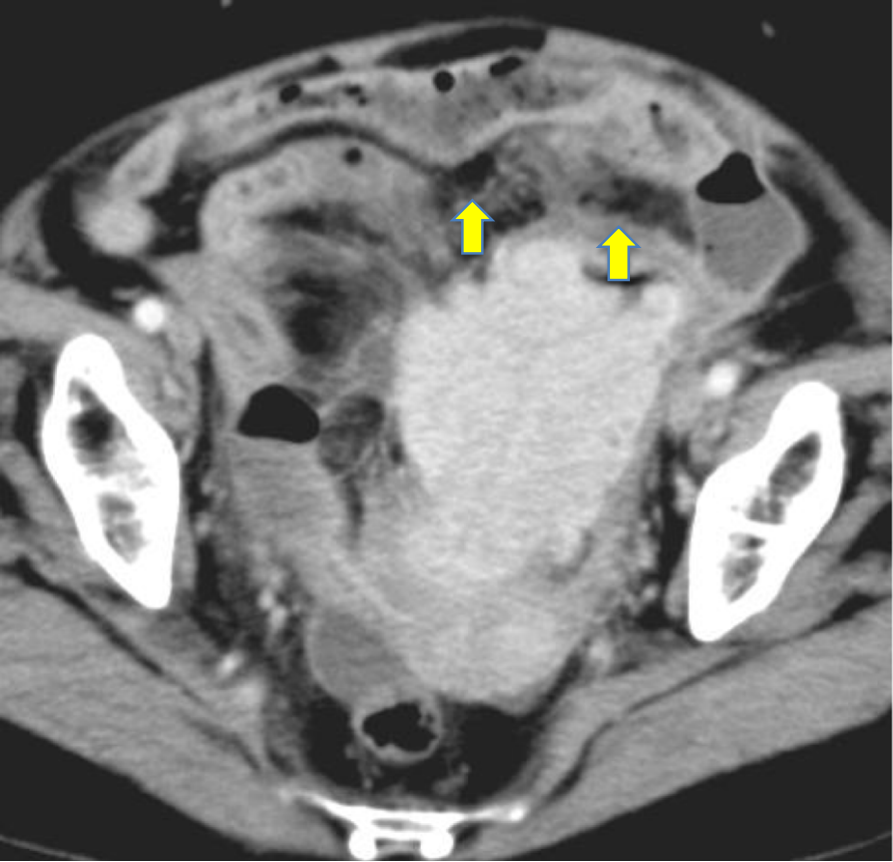
Plain abdominal computed tomography. Huge tumor is present at the sigmoid colon, and free air (arrows) is seen around the tumor

**Fig. 2:**
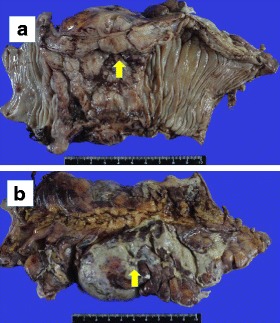
Macroscopic examination. **a** A type 1 tumor is present in the sigmoid colon. **b** The sigmoid colon is surrounded by contamination. Arrows indicate concavity suspected the site of perforation of the tumor

A 77-year-old woman with a 1-year history of lower abdominal pain and nausea was admitted to our hospital. Blood examination showed evidence of an inflammatory response (Table 1), and abdominal computed tomography revealed a sigmoid tumor with perforation (Fig. 1). We suspected sigmoid cancer with perforation, and the patient underwent emergency surgery. Open laparotomy revealed an extensive mass involving the sigmoid colon with surrounding contamination (Fig. 2). The abdominal mass was removed en bloc, including resection of the sigmoid colon. The abdomen was flushed to remove contamination. An artificial anus was made.

**Table 1:** Blood examination

Blood count		Biochemical parameters		Coagulation parameters	
WBC	8090/μL	TP	3.5 g/dL	PT (s)	14.3
RBC	360 × 10^4^/μL	Alb	1.59 g/dL	PT (%)	63.3
Hb	11.5 g/dL	T-bil	1.19 mg/dL	PT-INR	1.21
Plt	21.4 × 10^4^/μL	AST	28 IU/L	APTT (s)	45.4
		ALT	18 IU/L	ATIII	49%
		ALP	127 IU/L	FDP	40.0 *μ*g/mL
		LDH	219 IU/L	d-dimers	18.20 ng/mL
		*γ*-GTP	14 IU/L		
Tumor markers		BUN	15.6 mg/dL		
CEA	1.6 ng/mL	Cr	0.4 mg/dL		
CA19-9	5.2 U/mL	Na	134 mEq/L		
		Cl	3.6 mEq/L		
		CRP	29.49 mg/dL		

*WBC* white blood cells, *RBC* red blood cells, *Hb* hemoglobin, *Plt* platelets, *CEA* carcinoembryonic antigen, *CA19-9* cancer antigen 19-9, *TP* total protein, *Alb* albumin, *T-bil* total bilirubin, *AST* aspartate transaminase, *ALT* alanine transaminase, *ALP* alkaline phosphatase, *LDH* lactate dehydrogenase, *γ-GTP* gamma glutamyl transferase, *BUN* blood urea nitrogen, *Cr* creatinine, *Na* sodium, *Cl* chloride, *CRP* C-reactive protein, *PT* prothrombin time, *PT-INR* prothrombin time–international normalized ratio, *APTT* activated partial thromboplastin time, *ATIII* antithrombin III, *FDP* fibrin degradation products

**Fig. 3:**
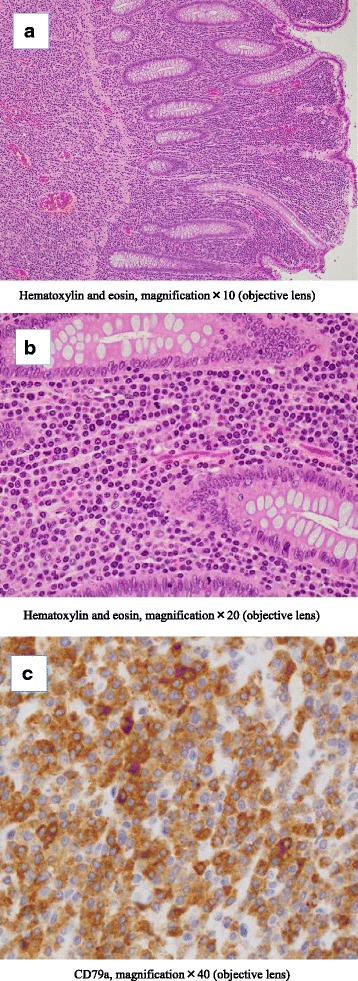
Microscopic examination. **a**, **b** Histopathologic examination of the resected tumor shows diffuse proliferation of atypical plasma cells (hematoxylin and eosin). **c** Immunohistochemical examination shows CD79a staining

**Fig. 4:**
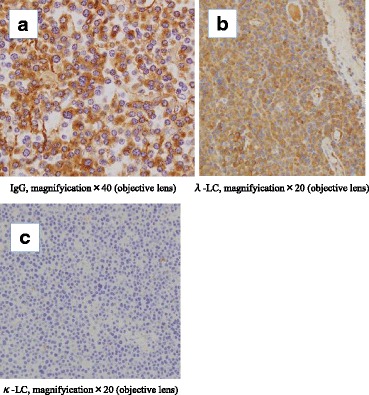
Immunohistochemical examination. **a**, **b** In situ hybridization shows that most of the tumor expresses immunoglobulin G and lambda light chain mRNA. IgG, immunoglobulin G; λ-LC, lambda light chain. **c** In contrast, there is no expression of kappa light chain mRNA. κ-LC, kappa light chain

Histopathologic examination showed that the oval mass was composed of a diffuse proliferation of plasma cells (Fig. 3a). At the concavity of the site of the perforation showed the tumor cell infiltrated into the subserosa and necrosis of tissue. But we were unable to identify the site of the perforation pathologically. The surgical margins were free from tumor cells. Immunohistochemical examination revealed positivity for CD79a (Fig. 3b), immunoglobulin G, and lambda light chain (Fig. 4a, b). Other markers (CD10, CD20, and kappa light chain) were negative (Fig. 4c). Pathological examination led to a diagnosis of plasmacytoma of the colon. The patient underwent bone marrow biopsy and bone imaging to exclude associated multiple myeloma. Her peripheral blood smear, serum protein electrophoresis, and urine immunoelectrophoresis for Bence-Jones protein were normal.

Postoperatively, the patient was discharged without any complications. She did not undergo postoperative adjuvant chemotherapy and has had no recurrence in 14 months of regular follow-up.

### Discussion

Extramedullary plasmacytoma accounts for only 3 to 5% of all plasma cell diseases. These tumors may be solitary or may precede, accompany, or follow the onset of multiple myeloma. Solitary extramedullary plasmacytoma has rarely been reported, and its natural history and diagnosis are unclear. Diagnosis of solitary extramedullary plasmacytoma requires the exclusion of associated multiple myeloma, which is determined by the absence of Bence-Jones protein in the urine, normal serum electrophoresis, and normal bone marrow biopsy [4]. Our present case met these criteria.

Alexiou et al. [5] reported that extramedullary plasmacytoma most often occurs in the nasopharynx or upper respiratory tract (82.2%). Only 17.8% of cases involve the gastrointestinal tract. The stomach and small intestine are the most commonly involved sites in the gastrointestinal tract. Primary isolated extramedullary plasmacytoma of the colon is extremely rare, occurring in only 0.028% of cases [5]. Therefore, its clinical features and prognosis are not well known.

The clinical presentation of extramedullary plasmacytoma of the colon is variable and may include abdominal pain, intestinal bleeding, and diarrhea. Gabriel and Savu [6] reported a rare case in which an extramedullary plasmacytoma was found with ileocecal junction perforation secondary to colonoscopic injury. This is the only previous report to describe extramedullary plasmacytoma with gastrointestinal perforation (Table 2).

**Table 2:** Well-documented cases of plasmacytoma of the colon

Author/year	Sex	Age (years)	Location	Clinical features	Therapy
Vasiliu and Popa/1928	F	47	Sigmoid	Anorexia, epigastric pain, glandular enlargement	?
Brown and Liber/1939	M	57	Colon, rectum	Rectal discomfort	?
Hampton and Gandy/1957	F	43	Rectum	Rectal pain and bleeding	Rectosigmoid resection
Miller/1970	M	35	Cecum	Anemia	Right hemicolectomy
William/1970	M	84	Cecum	Anemia	Right hemicolectomy
Neilson/1972	F	82	Sigmoid	Pain	Resection
Wing/1975	F	82	Ascending colon	Pain	Right hemicolectomy
Shaw/1976	F	47	Cecum	Diarrhea	Resection
Staples/1977	M	61	Sigmoid	Incidental operative finding	Resection
Daniel/1977	M	21	Descending colon	Pain, nausea, vomiting	Left hemicolectomy
Allion/1977	M	61	Sigmoid	None	Sigmoid colectomy
Adekunle/1978	M	35	Cecum	Pain	Right hemicolectomy
Terrence/1982	F	20	Transverse colon	Pain, rectal bleeding	Transverse colon resection
Sidani/1985	M	52	Sigmoid	Pain,	Resection
				rectal bleeding	
Rechard/1987	M	77	Cecum	Weight loss, anemia, pain, fecal occult blood	Right hemicolectomy
Saverio Ligato/1996	M	45	Hepatic flexure of the colon	Anemia	Extended right hemicolectomy
Holland/1997	M	62	Sigmoid colon	Pain	Sigmoid colectomy
Lattuneddu/2004	M	86	Sigmoid colon	Pain, rectal bleeding, asthenia	Segmental resection of the left colon
Gupta/2007	M	42	Diffuse colon	Diarrhea	Subtotal colectomy
Jones/2008	M	65	Sigmoid colon	Dysuria, abdominal pain	Sigmoid colon resection
Jone/2008	M	57	Sigmoid colon	Fatigue, melena	Hartmann resection
Doki/2008	M	64	Ascending colon	Pain	Right hemicolectomy, lymph node dissection, excision of Gerota’s fascia, partial resection of the posterior portion of the liver
Collado Pacheco/2009	M	74	Right colon	Diarrhea, pain, rectal bleeding	?
Kodani/2011	M	42	Sigmoid	Fecal occult blood	Endoscopic submucosal resection
Nakagawa/2011	F	84	Cecum and rectum	Medical examination	Endoscopic submucosal resection
Lee/2013	M	45	Transverse colon	Pain	Extended left hemicolectomy
Zihni/2013	M	54	Descending colon	Pain and weakness	Left hemicolectomy and small intestinal resection
Han/2014	M	49	Transverse colon	Pain	Left hemicolectomy
Emmanuel/2014	M	62	Cecum	Perforation during diagnostic colonoscopy	Right hemicolectomy
Parnel/2015	F	72	Right colon	Fatigue, light-headedness, dyspnea, dark stool	Right hemicolectomy Distal ileal resection

*F* female, *M* male

In the present case, we were unable to determine the cause of the perforation by pathologic examination. We consider that the tumor was necrosed and perforated; otherwise, as the tumor grew, the intestinal internal pressure increased, resulting in perforation of the sigmoid colon.

Postoperative chemotherapy has no effect on the course of extramedullary plasmacytoma. Our patient did not undergo postoperative adjuvant chemotherapy, and she has had no relapse to date. However, careful follow-up is required.

Because primary isolated extramedullary plasmacytoma in the colon is very rare, the clinical course, treatment guidelines, and prognosis remain unclear. Further study of the clinical features of primary isolated extramedullary plasmacytoma of the colon is necessary to ensure that adequate treatment is administered.

## Conclusions

We have described a rare case of extramedullary plasmacytoma of the gastrointestinal tract with perforation of the sigmoid colon. In this case, the prognosis was good because of appropriate treatment involving early surgery.
